# Considering the Node Level in Error Correction for DMFBs †

**DOI:** 10.3390/mi16091013

**Published:** 2025-08-31

**Authors:** Koki Suzuki, Shigeru Yamashita, Hiroyuki Tomiyama, Ankur Gupta

**Affiliations:** 1 Graduate School of Information Science and Technology, Ritsumeikan University, Ibaraki 567-8570, Osaka, Japan; is0562xf@ed.ritsumei.ac.jp; 2 College of Information Science and Technology, Ritsumeikan University, Ibaraki 567-8570, Osaka, Japan; 3 College of Science and Engineering, Ritsumeikan University, Kusatsu 525-8577, Shiga, Japan; ht@fc.ritsumei.ac.jp; 4 Department of Computer Science and Engineering, Netaji Subhas University of Technology, New Delhi 110078, India; agupta4@cs.iitr.ac.in

**Keywords:** DMFB, division error, target node, dilution graph

## Abstract

In recent years, a type of biochip known as a Digital Microfluidic Biochip (DMFB) has been actively researched in the field of life sciences. DMFBs perform dilution operations by mixing reagent solutions and buffer solutions at a 1:1 ratio to generate droplets with the desired concentration. One of the challenges of DMFBs is that droplets may not always be evenly split during the droplet division process. To address this issue, an error correction method utilizing error cancellation has been proposed. This method modifies the dilution graph to minimize the impact of division errors on the target node. However, this approach has a significant drawback: when large division errors occur in nodes close to the target node, they can introduce substantial concentration errors at the target node. In this paper, we propose a method that duplicates nodes near the target node and performs re-dilution to correct errors. Furthermore, we present an efficient and accurate error correction approach by modifying the dilution graph so that the output nodes of the dilution operation are at equal levels relative to the target node. Through simulations conducted 10,000 times, we demonstrate that our method effectively reduces the average concentration error at the target node.

## 1. Introduction

In recent years, various biochips have been studied. Biochips are devices that allow complex biochemical assays such as point-of-care diagnostics (e.g., malaria, SARS-CoV-2), blood glucose monitoring for diabetes, DNA analysis, PCR-based genetic testing, protein crystallization and drug discovery, etc. [1]. DMFB (Digital MicroFluidic Biochip (DMFB)) is a kind of biochip [2,3,4], which has electrodes arranged in a two-dimensional array. By electrostatic induction, these electrodes can mix, dilute, transfer, and split droplets on the chip, making DMFBs ideal for biochemical experiments [4,5]. A DMFB can, for example, mix reagent droplets and buffer solution at a 1:1 ratio and perform multiple dilution operations to generate droplets of a desired concentration.

One of the problems of DMFBs is that the droplets are not evenly split after the dilution process. This is because one droplet has a larger volume than the other. As a result, the final droplets produced are affected in the same way, resulting in droplets that differ from the target concentration [6,7]. As a countermeasure for errors in droplet splitting, there is a method to correct errors by deforming the dilution graph without performing the re-dilution operation [8]. This method can further reduce the effect of errors in dilution graphs created by the REMIA method [9] by taking into account the combination of nodes when there are multiple intermediate droplets. However, this method can only deal with errors at the node where the combination is considered, and if a large error occurs at a node close to the node where the final droplet is located (hereinafter referred to as the target node), the target node will have a large error. In this paper, we propose a method for the re-dilution of nodes close to the target node. In the resulting dilution graph, error correction is performed by re-diluting the nodes that are close to the target node and re-diluting the droplets at those nodes. As a result, even when errors occur at nodes close to the target node, the impact on the target node can be minimized. In addition, the impact of the error can be further reduced by considering the level of the intermediate droplet output destination node from the target node during the dilution graph deformation. In the dilution graph created by the proposed method, it is shown that the average concentration error of the target concentration can be reduced by about 8% and the maximum concentration error can be reduced by about 16% compared to existing methods.

This paper consists of five sections. Section 2 describes the fundamentals of DMFB. Section 3 describes the deformation of the dilution graph in the proposed method. Section 4 presents experimental results and discussion. Section 5 presents future issues and the conclusion of this paper.

## 2. Preliminaries

This section describes the basics of DMFBs (Digital MicroFludic Biochips). First, the architecture of DMFBs is explained. Next, we explain the dilution and partitioning operations of DMFBs and the partitioning errors that occur during the droplet operation. Then, we introduce REMIA, a dilution graph generation method that minimizes the amount of reagents used, and finally, we introduce a method by Wada et al. [10], which is one of the existing countermeasures against division errors.

### 2.1. DMFB (Digital MicroFluidic Biochip)

#### 2.1.1. Architecture

**Figure 1:**
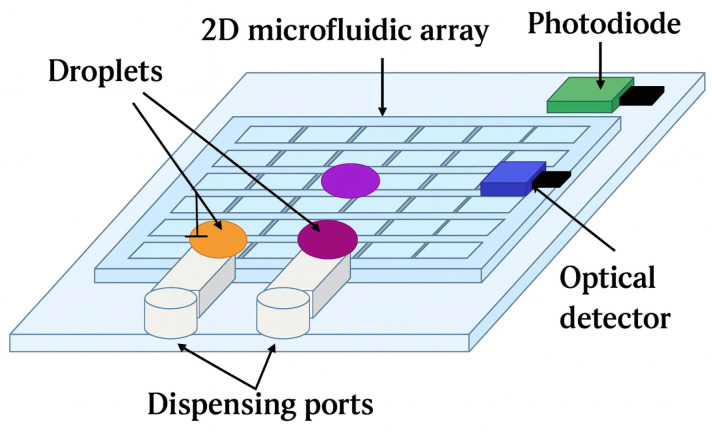
A conceptual diagram of the DMFB architecture.

The DMFB is a biochip that uses electrodes placed on a two-dimensional array and electrostatic induction to manipulate droplets. Figure 1 shows the architecture of the DMFBs [2,3,4]. Reagent solution or buffer solution is injected through the dispensing ports in the figure. Then, droplets are moved by applying voltages to electrodes arranged in a two-dimensional array. The voltage changes surface tension (electrowetting effect), which causes droplets to move, split, merge, or mix on the chip. In DMFBs, photodiodes are typically used to monitor the contents of droplets, such as detecting fluorescence or absorbance from biochemical reactions, while optical detectors are mainly employed to sense the presence and position of droplets, ensuring that droplet operations are correctly executed.

#### 2.1.2. Dilution and Splitting Operations

**Figure 2:**
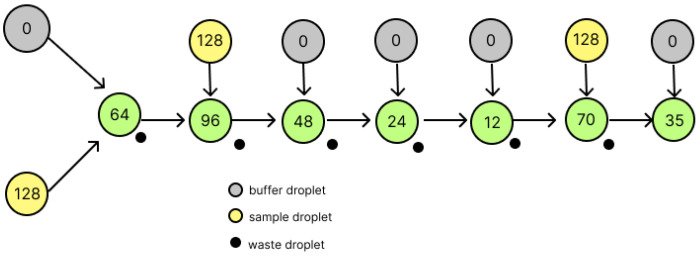
Dilution and splitting of DMFBs.

In DMFBs, the reagent solution and buffer solution are mixed 1:1 and the solution can be divided 1:1. As an example, the operation to generate droplets with a concentration of 35/128 is shown in Figure 2. The yellow circle labeled 128 represents a reagent droplet, the gray circle labeled 0 represents a buffer solution, the black circle represents a waste droplet generated after splitting, and each green circle represents an intermediate droplet. All nodes have only the numerator of the concentration where 128 is the denominator. By repeating the mixing and splitting operations in this way, droplets of the target concentration can be generated.

#### 2.1.3. Splitting Error

Even if a DMFB is perfectly made, unbalanced splitting may still occur due to uncontrollable factors. Even worse, it appears randomly and its effect is completely unpredictable [11]. Thus, in a DMFB, the droplet volume of one droplet may be larger than the volume of the other droplet, and the volume of the other droplet may be smaller than the volume of the first droplet [6,12]. Unbalanced splitting affects not only the volume of the present output droplets but also the concentration of the output droplet of the next mix operation [11].

**Figure 3:**
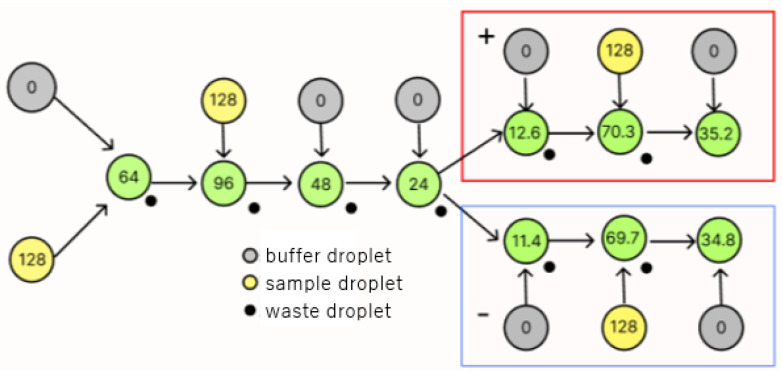
DMFB splitting error.

As an example, Figure 3 shows the flow of operations when a splitting error occurs in a dilution graph that produces droplets with a concentration of 35/128. In Figure 3, the fourth dilution operation produces a 5% division error. In Figure 3, the paths marked with red squares show the results when the larger-volume droplet produced by the splitting error is used, while the paths marked with blue squares show the results when the smaller-volume droplet is used. Compared to the original target concentration of 35/128, an error of 0.2/128 is observed. In the case shown in Figure 3, only one splitting error occurs, but there may be cases where splitting errors occur at more than one node. The splitting errors that can occur in DMFB dilution splitting can be corrected or canceled out by remixing both of the droplets produced by it later, and a number of methods have been proposed to counteract splitting errors using this technique [6,7,8]. However, these increase the run time and droplet usage, and it is not efficient to remix each time an error occurs.

### 2.2. REMIA

**Figure 4:**
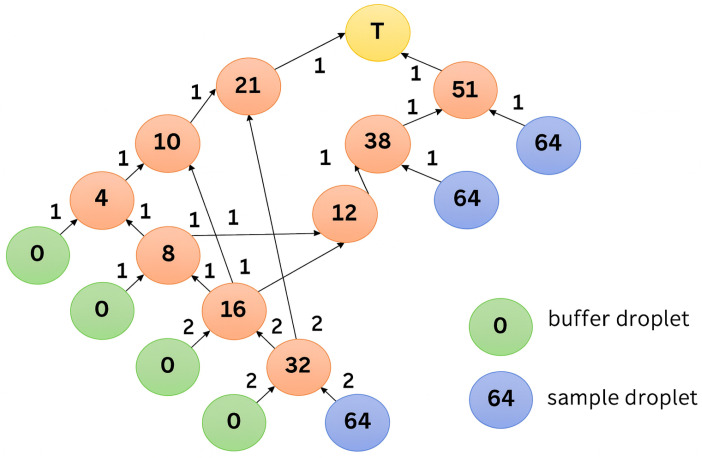
Example of a dilution graph created by REMIA.

**Figure 5:**
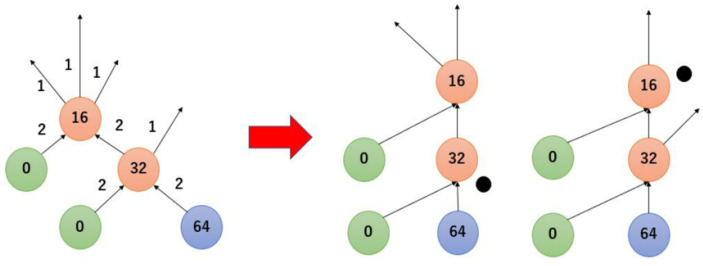
Detailed image when more than 3 drops are handled at the same time.

Several dilution graph algorithms have been developed for DMFBs [11,13,14], and one of them, REMIA [9], is an algorithm that can generate dilution graphs with small amounts of reagents. Therefore, due to the nature of the algorithm, it is highly likely that droplets of equal concentration will be generated at multiple locations. The dilution graph generated by REMIA is shown in Figure 4. The number on the node represents the numerator of the concentration, with 64 as the denominator, and the number next to the arrow represents the number of droplets. The node marked T is the target node. Around the 32/64 and 16/32 nodes, it looks as if four droplets are generated and two droplets are moved at each edge, but as described in Section 2.1, DMFBs can only mix one drop at a time to generate two drops. In the actual operation, the droplets are generated at two different locations as shown in Figure 5.

### 2.3. A Countermeasure Method for Division Errors by Wada et al.

**Figure 6:**
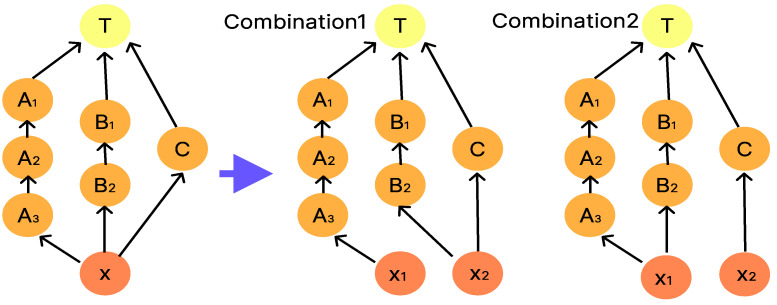
Examples of how to combine intermediate droplets.

As a countermeasure against splitting errors, the method proposed in [9] minimizes the impact of splitting errors on the droplets at the target node by selecting the optimal combination of intermediate droplets in the dilution graph generated by REMIA, when there are multiple intermediate droplets. An example is shown in Figure 6. In the dilution graph on the left side of the arrow in Figure 6, one drop moves from node X to the three nodes A, B, and C, but as shown in Figure 5, DMFB can only perform a 1:1 dilution partitioning operation, so the three drops at node X must be divided into two drops, one and two, as shown in the two dilution graphs on the right side of the arrow. The selection of the combination is also made with consideration for the dilution graph to be the one least affected by the error. Droplets with concentration errors caused by segmentation errors are referred to as “error droplets” in the following.

**Figure 7:**
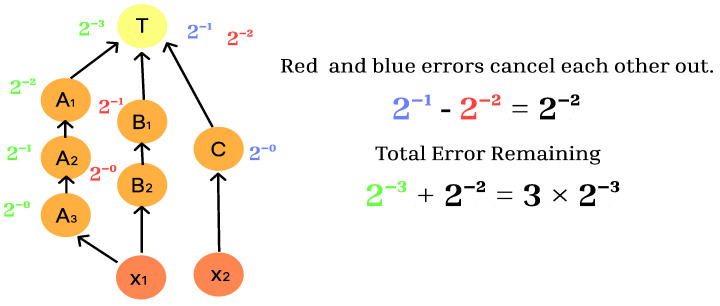
Intermediate droplet combination 1.

In this method, the selection of intermediate droplet combinations is made by focusing on the total amount of error droplets remaining. The remaining error droplet volume in the dilution graph for combination 1 in Figure 6 is shown in Figure 7. Each dilution operation splits the droplet by 1/2, so that the droplet split at node X uses 1/8 of the droplet fraction at the target node. If a splitting error occurs at node X, 1/8 of the error droplets remain as they are. On the other hand, of the droplets split at node Y, 1/4 of the droplets through node B are used at the target node. The droplet through node C is used in 1/2 of the error droplets. If a splitting error occurs at node Y, the errors in the droplet through node B and the error in the droplet through node C cancel out, leaving 1/4 of the error droplet. The total amount of error droplets remaining is 1/8 of the error droplets from node X and 1/4 of the error droplets from node Y, which is 3/8.

**Figure 8:**
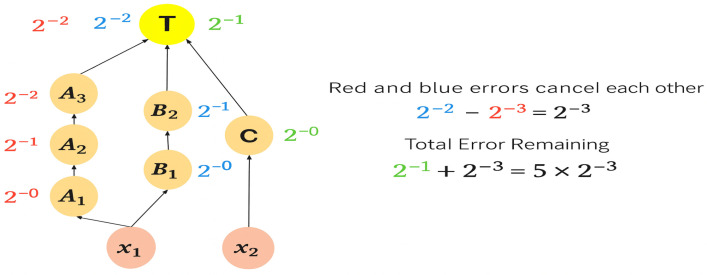
Intermediate droplet combination 2.

Next, the remaining error droplet volume in the dilution graph for combination 2 in Figure 6 is shown in Figure 8. If a splitting error occurs, the errors in the droplets split at node X that went through node A and those through node B are canceled out, leaving 1/8 of the error droplets. Droplets split at node Y are used in a 1/2 ratio at the target node. If a splitting error occurs, 1/2 of the error droplets remains as it is. Therefore, the total amount of error droplets remaining is 5/8, which is the sum of 1/8 of the error droplets from node X and 1/2 of the error droplets from node Y. The impact of the error is smaller when the total remaining amount of Ler droplets is smaller. Therefore, in Figure 6, the dilution graph of combination 1 is selected and the dilution graph is transformed. However, this approach can only address errors that occur at the node where the combinatorial selection was made. Another drawback is that if a large error occurs at a node close to the target node, the large error at the target node cannot fully cancel out the division error and the error remains.

## 3. Error Correction Techniques Based on Node Redundancy with Node Level Consideration

This section describes the proposed method of correcting partitioning errors through node doubling and dilution graph deformation considering the node level.

### 3.1. Overview of the Proposed Method

The proposed method in this paper consists of two ideas based on the method of Wada et al. and its problems described in Section 2.3. The first is error correction in remixing by node doubling, and the second is error cancellation by dilution graph deformation considering the node level. In the dilution graph generated by REMIA, the proposed method duplicates the nodes close to the target node, which is difficult to deal with using Wada et al.’s method, and later performs a remixing operation to correct errors. In addition, if a node has more than one destination node (two is the maximum for DMFB) during dilution graph transformation, By deforming the dilution graph so that their levels from the target node are equal, if a splitting error occurs, the error droplets are canceled just before they reach the target node, and the error is corrected. Section 3.2 discusses node duplication, and Section 3.3 discusses graph deformation considering the node level.

### 3.2. Node Redundancy

**Figure 9:**
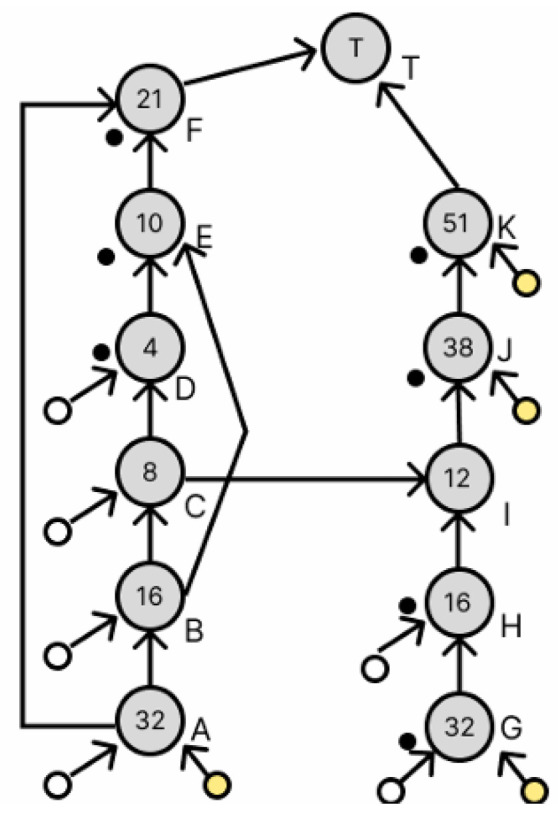
Dilution graph applying the proposed method.

This section describes node redundancy for error correction by remixing. In the dilution graph in Figure 9, the yellow circle represents the reagent droplet, the white circle represents the buffer droplet, the black circle in the lower left corner of each node represents the waste droplet, and the number in each node represents the numerator of the concentration whose denominator is 64. Consider the case of duplexing node F in the dilution graph in Figure 9. In this section, we describe two methods of duplication: simple duplication and duplication using discarded droplets.

#### 3.2.1. Simple Redundancy

**Figure 10:**
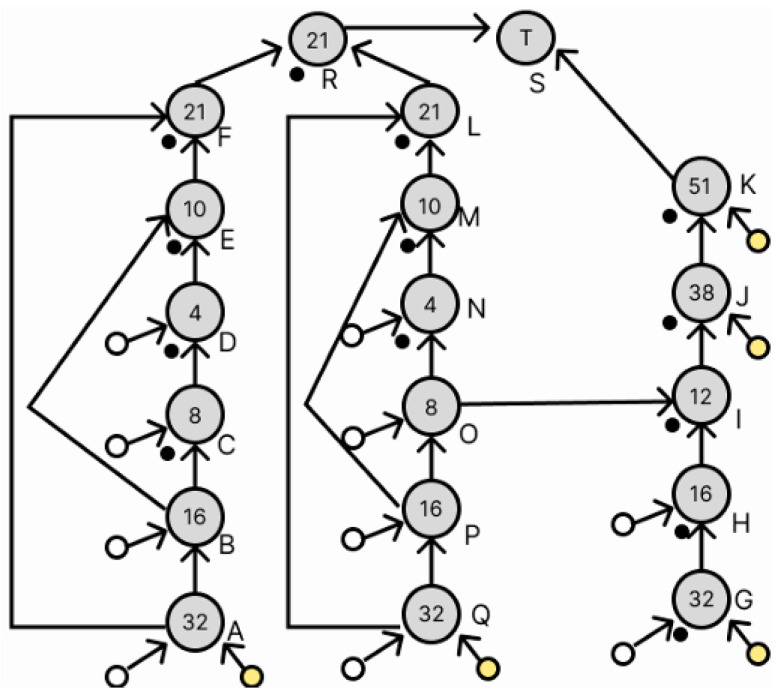
Dilution graph applying the proposed method.

The dilution graph for a simple duplication of node F is shown in Figure 10. The path from node A to node F is duplicated to produce droplets of the same concentration as node F. At node L, droplets of the same concentration as those at node F are generated, and these are remixed at node R to correct the error. However, this method duplicates the path and all of its nodes, which can significantly increase operation time and droplet usage.

#### 3.2.2. Redundancy Using Waste Droplets

This section describes node redundancy using waste droplets. As shown in Figure 10, a simple duplication of node F requires an extra droplet. However, since the dilution graph generated by REMIA is realized with a small number of droplets, simply doubling node F is not enough to generate droplets of the same concentration. Therefore, we use the discarded droplets to duplicate the node and finally allow the duplication of node F. Figure 9 shows that the discarded droplets are located at nodes D, E, F, G, H, J, and K. First, node E is redundant, and then node F is redundant.

**Figure 11:**
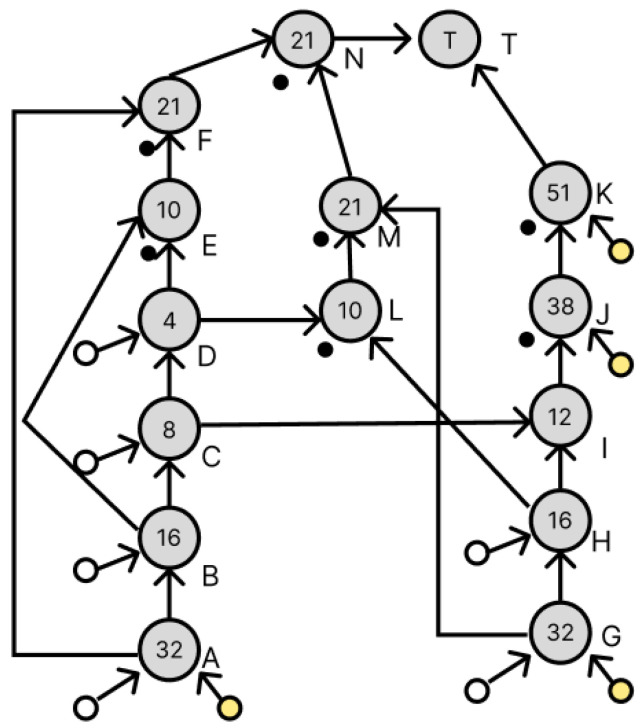
Dilution graph with node redundancy using waste droplets.

**Figure 12:**
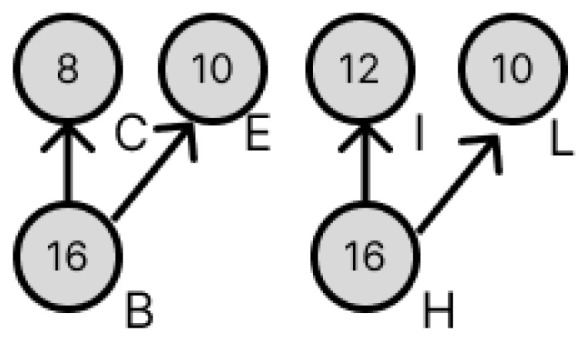
Output destination for droplets of concentration 16/64.

Figure 11 shows the dilution graph with node E being redundant. The areas indicated in red have been changed. Since there is a droplet of concentration 10/64 at node E, redundancy at node E requires the creation of a droplet of the same concentration. To generate a droplet with a concentration of 10/64, a droplet with a concentration of 4/64 and a droplet with a concentration of 16/64 are required, which exist as a droplet at node D and a waste droplet at node H, respectively, and these are used to generate node L. Next, the dilution graph with redundant node F is shown in Figure 12. The red part has also been changed. To duplicate node F, we want to generate a droplet with a concentration of 21/64. This droplet is generated by a droplet with a concentration of 10/64 and a droplet with a concentration of 32/64. The 10/64 droplet exists in node L, which was generated earlier, and the 32/64 droplet exists as a waste droplet in node G. We can use these to generate a droplet with a concentration of 21/64 as node M. We can now generate a droplet with a density of 21/64 as node M. Node N is added to perform the remixing operation of the droplets at node F and node M generated here. This redundant method did not increase the number of drops used and the number of drops discarded despite the addition of a node for remixing.

### 3.3. Transformation of Dilution Graphs Considering Node Level

**Figure 13:**
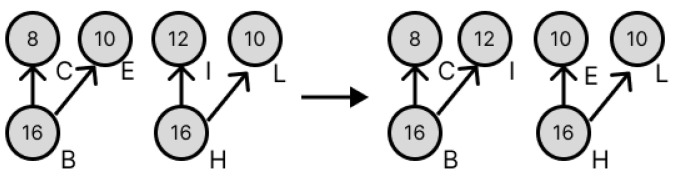
Combination of output destination nodes considering the level from the target node.

**Figure 14:**
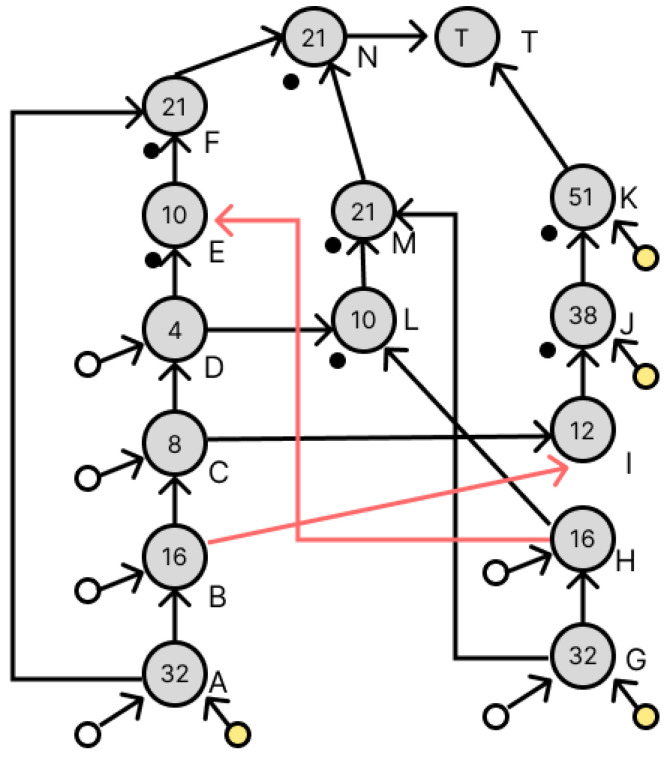
Dilution graph considering the level of nodes E and L from the target node.

This section describes how to transform the dilution graph considering the node level. As mentioned in Section 2.3, the error droplets produced by a splitting error can be canceled out by later mixing both of the droplets with concentration errors to cancel out their respective volume errors. Therefore, when there are multiple output destinations for a node, these are redundant so that their levels are equal in terms of the target node, and the difference in the amount of error droplets remaining in each path is reduced, thereby reducing the total amount of error droplets remaining when they are canceled out. Consider nodes B and H in Figure 11. Nodes B and H both have a droplet of concentration 16/64, which is used by four drops in the dilution graph. Figure 12 shows the destination nodes of nodes B and H, respectively. Here, from Figure 11, node L is a node created by the redundancy of node B, so its level from the target node is equal. If, as in Figure 13, the node to redundancy and the droplets of the input of the node generated by it are output from different nodes, transform the dilution graph so that they are output from the same node. In Figure 14, this is the result of transforming the dilution graph by considering the level from the target node for nodes E and L. The area indicated by the red arrow has been changed.

## 4. Experimental Results

We compared the error rates of concentration errors at the target node for these three dilution graphs: a dilution graph pre-deformed by the method of Wada et al. (Figure 9), a dilution graph deformed by applying the node redundancy method using waste droplets (Figure 11), and a dilution graph deformed by considering the node level (Figure 14). The experiment was simulated 10,000 times by randomly generating splitting errors for all nodes based on the probability table shown in Table 1. The average and worst error values at the target node for the three dilution graphs are shown in Table 2.

## 5. Conclusions

In this paper, we propose a method to correct partitioning errors in DMFB by redundancy of nodes close to the target node and deformation of the dilution graph to consider the node level at which the droplet moves. The proposed method focuses on the nodes close to the target node that are likely to have a large impact on the target droplet, and performs re-dilution only on those parts, thereby reducing the impact of the error on the target node and generating a more accurate dilution graph. In the experiments in this paper, we compared graphs deformed by the proposed method with dilution graphs deformed by the method of Wada et al. The dilution graph deformed by the proposed method showed the best results for both the worst error value and the average error value of the target node in the case of randomly generated errors.

However, this method involves the addition of nodes and droplets that were not originally present, in order to carry out the redundancy of nodes and dilution graph deformation for node level considerations. Also, due to the redundancy characteristic of this method, the number of operations to be performed and the execution time may increase even for the part of the path that is redundancy alone. In addition, in this paper and in the present experiment, error correction was performed by making changes only to the nodes close to the target node, but it is possible that the proposed method will give better results for other nodes as well. Therefore, the impact of a single node on the target node when an error occurs can be known using a measure called RRE [9], which indicates the residual error for each node. Future issues include narrowing down the number of nodes to be duplicated or added, and considering the trade-off between error tolerance and the number of droplets used. Lastly, we consider it interesting to check how our method can indeed deal with splitting errors for experiments on a real device.

## Figures and Tables

**Table 1:** Probability table for split errors [11].

Percentage of Split Errors	Frequency
0%	49.5%
1%	32.2%
2%	13.7%
3%	3.8%
4%	0.7%
5%	0.1%

**Table 2:** Average and worst error values at the target nodes of the three dilution graphs.

	Average Error	Worst Error	Number of Drops Used	Number of Dilution Operations
Figure 9 (Wada et al. Method)	0.0000467146%	0.0202777646%	10	12
Figure 11 (Redundancy with Waste Droplets)	0.0000317079%	0.0187978745%	10	15
Figure 14 (Node Level Consideration)	0.0000073830%	0.0157602094%	10	15

## Data Availability

The original contributions presented in this study are included in the article. Further inquiries can be directed to the corresponding author.
